# Remarkable response to pazopanib plus vivolumab in a patient with pericardial synovial sarcoma carrying a novel genotype BRCA2 c.968dupT: A case report

**DOI:** 10.1111/1759-7714.15237

**Published:** 2024-02-07

**Authors:** Xing Zhang, Qinqin Xu, Yongchang Zhang

**Affiliations:** ^1^ Graduate Collaborative Training Base of Hunan Cancer Hospital, Hengyang Medical School University of South China Hengyang Hunan China; ^2^ Lung Cancer and Gastrointestinal Unit, Hunan Cancer Hospital, Department of Medical Oncology The Affiliated Cancer Hospital of Xiangya School of Medicine Changsha Hunan China; ^3^ Department of Medical Oncology Qinghai Provincial People's Hospital Xining Qinghai China

**Keywords:** BRCA2, immunotherapy, pericardial synovial sarcomas (PSS), tyrosine kinase inhibitor (TKI)

## Abstract

Pericardial synovial sarcomas (PSS) have a low incidence rate and are highly invasive with a dismal prognosis. Standard treatment includes surgery, radiotherapy and chemotherapy but with limited response. Here, we report the case of a 15‐year‐old nonsmoking youngster diagnosed with PSS who developed disease relapsed from surgery after 1 month. Next‐generation sequencing (NGS) using baseline tissue was performed, and BRCA2 c.968dupT was detected. Then pazopanib (a multitargeted inhibitor) plus nivolumab (an immune checkpoint inhibitor) was administered, with a partial response and progression‐free survival of 14 months. BRCA2 c.968dupT has not previously been reported in PSS and its response to targeted combination immunotherapy are not well characterized. Here, we report the efficacy of pazopanib combined with nivolumab in a PSS patient harboring BRCA2 c.968dupT and also provide the clinical evidence of the utility of NGS in exploring actionable mutations for solid tumor. Combination therapy based on immunotherapy may be a potential treatment choice for PSS harboring BRCA2 mutation.

## Graphical abstract

A huge solid cystic mass located in the mediastinum was found in a 15‐year‐old youngster suffering shortness of breath. The synovial sarcoma was diagnosed by tissue of emergency surgery. One month later, the mediastinum lesion of patient relapsed. The next‐generation sequencing was performed and revealed BRCA2 c.968dupT mutation. Eventually, the pazopanib plus nivolumab was administered with progression‐free survival of 14 months.

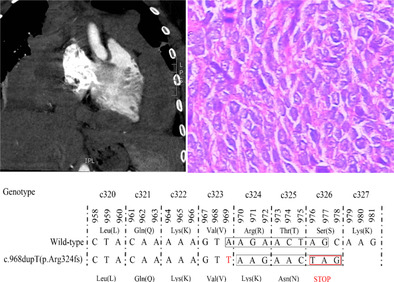

## INTRODUCTION

Synovial sarcoma (SS) is a rare malignancy of mesenchymal origin, accounting for approximately 8%–10% of all soft tissue sarcomas.^1^ It most commonly affects young adults between the ages of 15 and 30 years. SS can occur in almost any anatomic site, and more than 80% originate in the deep soft tissues of the extremities. However, it occasionally occurs in rare sites such as the head and neck, lungs, chest wall, mediastinum, orbit, and esophagus, and pericardial SS has been reported rarely.^2^ The diagnosis of SS is based on histology and molecular biology tests including t(X;18) chromosomal translocation confirmed by fluorescence in situ hybridization (FISH) or reverse transcription‐polymerase chain reaction (RT‐PCR). The treatment of PSS varies, but usually involves surgery, radiotherapy, and chemotherapy. Despite these multimodal treatment options, the prognosis for patients with synovial sarcomas remains poor, with an expected 5‐year survival in adult patients ranging from 50% to 60%.^3^

Pazopanib is an oral, multitargeted tyrosine kinase inhibitor (TKI) that predominantly inhibits vascular endothelial growth factor receptors 1, 2, and 3. Pazopanib is approved for the treatment of soft tissue sarcoma (STS), which have some activity in synovial sarcomas. In the NCT00753688 study, pazopanib was administered to 369 patients with metastatic soft‐tissue sarcoma after failure of standard chemotherapy. Progression‐free survival (PFS) and significant improvement was observed with pazopanib compared with a placebo (4.6 vs. 1.6 months).^4^ In a retrospective Japanese study, patients with SS received pazopanib and the PFS was 16.4 weeks.^5^ Immunotherapy has led to a breakthrough in treatment with approval as first‐line treatment by the FDA due to a robust response, including lung cancer, head and neck carcinoma and breast cancer. The efficacy of PD‐1 inhibitors (nivolumab and pembrolizumab) has been also investigated in SS. The SARC028 study revealed that pembrolizumab showed encouraging activity in patients with soft tissue sarcoma, with an mPFS of 4.2 months, 18% of responses and a 6‐month progression‐free survival rate (PFSR) of 32%.^6^ Similar results were observed also in the NCT03277924 registration study. The combination of antiangiogenic agents and nivolumab was investigated in patients with advanced soft tissue sarcoma, with an mPFS of 5.6 and a 6‐month PFSR of 48%.^7^

Breast‐cancer susceptibility gene 2 (BRCA2) is normally involved in the sophisticated DNA repair process, especially the homologous recombination pathway of double‐strand break (DSB) repair mechanisms. It is a tumor suppressor gene, and the most common mutations in BRCA2 are shift and missense mutations, the mutant phenotypes of which predispose individuals to breast and ovarian cancers. Chemotherapy, immunotherapy, or targeted therapy is commonly administered in cancer patients harboring BRCA2 mutation.^8^, ^9^, ^10^ The response of nivolumab in BRCA‐mutant recurrent ovarian cancer is robust and in a previous study the objective response rate was 67%.^11^

Here, we present a patient with BRCA2‐mutant PSS who could benefit from pazopanib combined with nivolumab and achieved a partial response with PFS of 14 months.

## CASE REPORT

**FIGURE 1:**
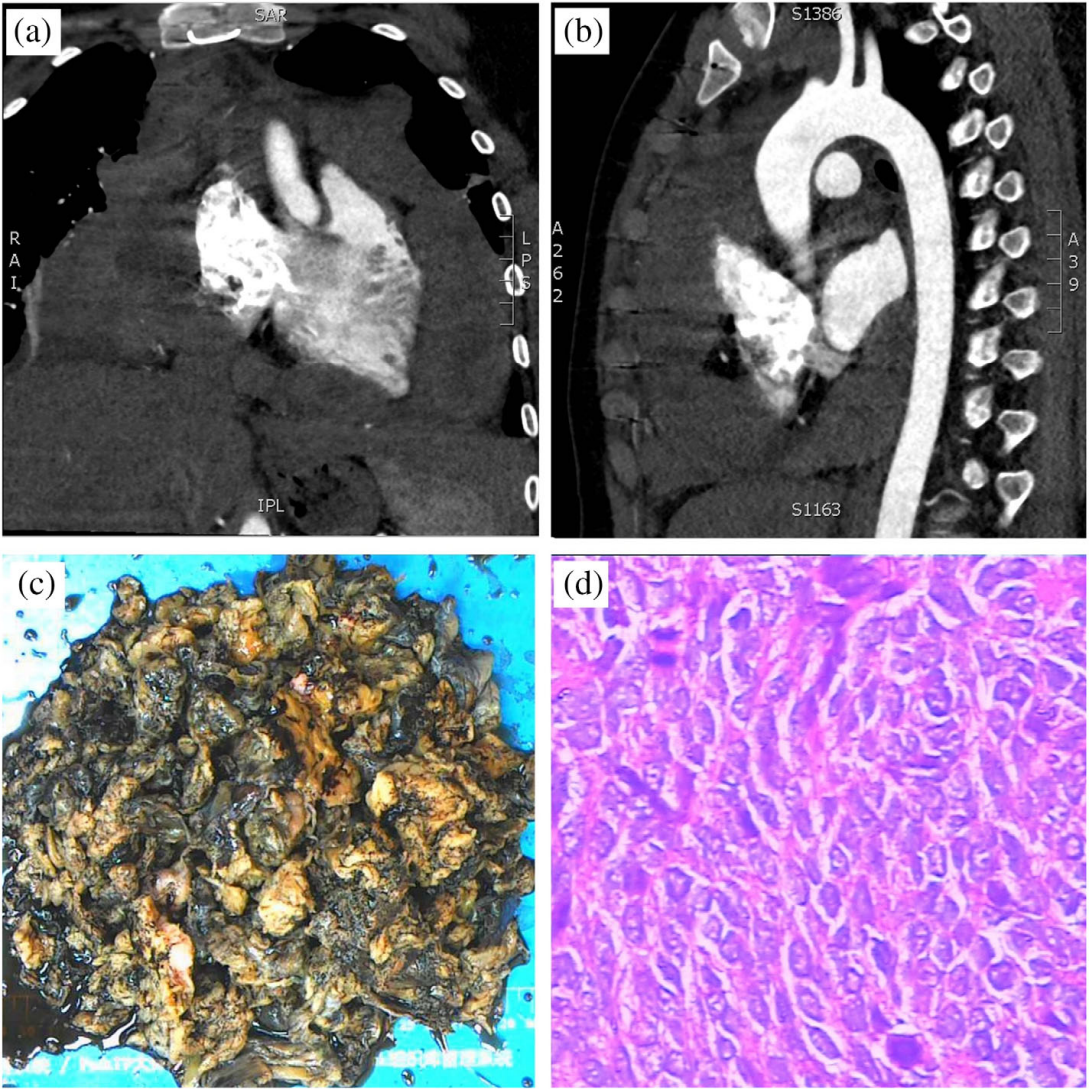
(a, b) Computed tomography (CT) of the chest and heart. (c) Gross examination of a huge solid cystic mass. (d) Hematoxylin and eosin stain of the solid cystic mass, ×400.

A 15‐year‐old nonsmoking youngster visited Hunan Cancer Hospital with a history of cough and shortness of breath for 2 weeks. His symptoms worsened over a few days. He had no history of trauma and tumor. No specific other symptoms were reported, and his blood tests were almost normal. Physical examination showed his heart rate was 110 beats per minute. No blood sputum was present. There was no fever, no shivering, and no lymphadenopathy. Color ultrasonography showed pericardial effusion. Results of emergency computed tomography (CT) revealed a huge solid cystic mass located in the mediastinum with pericardial effusion (Figure 1a,b). To avoid tamponade, the patient received emergency pericardium fenestration surgery. In the surgery, it was obvious to see the pericardium contained dark red pericardial effusions. An approximately 25 × 25 cm lump surrounded the aortic arch, ascending aorta, and pulmonary artery from the anterior superior mediastinum and covered the surface of the beating heart. The lumps grew downward into the pericardium and were adherent to the surface of the right ventricular outflow tract. The boundary of the mass was still clear, which was resected during the operation. Grossly, the fleshy mass had multinodular cysts, which were filled with dark red bloody fluid (Figure 1c). Histological examination revealed uniform spindle cells tightly arrayed with round and ovoid cell nucleus (Figure 1d), which was the most typical subtype of synovial sarcoma. Immunohistochemistry staining was negative for cytokeratin, epithelial membrane antigen (EMA), leukocyte common antigen (LCA), CK19, Syn, CD34, myoD1, myoglobin CD68, CK18, SMA, and S‐100 but positive for calponin, vimentin, Bcl2, CD99, CD56, and TLE. A large panel gene detection including 168 somatic gene mutations showed that the genotype of this patient was BRCA2 c.968dupT, ERBB3 p.T1225I, and MSH2 p.272 fs. The TPS score of PD‐L1 expression was 0 (22C3, Dako). Based on the clinical symptoms, gross findings, histological examination, and immunohistochemistry, the patient was diagnosed with pericardial synovial sarcoma.

**FIGURE 2:**
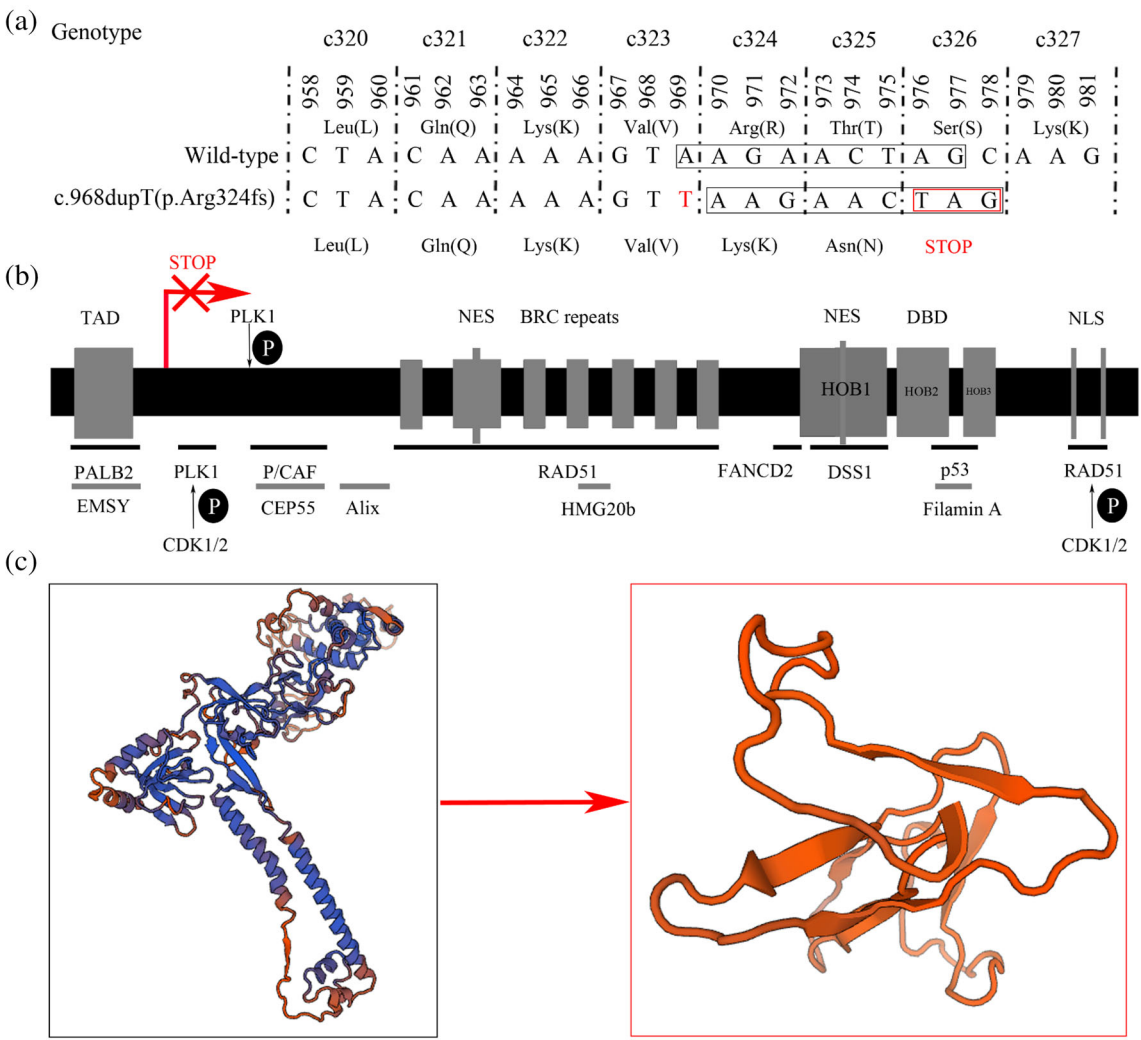
(a) Base change of BRCA2 c.968dupT. (b, c) Structure and functional domains of BRAC protein.

Results of base sequence analysis showed that, after inserting with a “T,” the whole gene was frameshift and transformed to be a new nonsense mutation, and we found that amino acid from code 326 to code 3419 was lost (Figure 2a). Protein analysis showed all the active peptides, including RAD51 binding site and functional domains were almost destroyed (Figure 2b,c).

Synovial sarcoma (SS) is a malignant mesenchymal neoplasm that occurs mainly in older children and young adults. A diagnosis of synovial sarcoma is based on histology and molecular biology tests including t(X;18) chromosomal translocation confirmed by FISH or RT‐PCR. The treatment of SS varies, usually involving surgery, radiotherapy, and chemotherapy.^3^ After progression, there are no standard treatments apart from those in clinical trials. Without standard adjuvant chemotherapy, positron emission tomography (PET)‐CT scan showed that the disease had relapsed after 1 month. Based on the pathology and genotype, pazopanib (GlaxoSmithKline, 800 mg, per os, once a day) combined with nivolumab (Bristol‐Myers Squibb, 3 mg/kg bodyweight, intravenously guttae, every 2 weeks) were initiated. A partial response was obtained after treatment for 6 weeks later with a chest CT evaluation. The adverse events were gray hair. The patient still had a consistent partial response for at least 14 months.

## DISCUSSION

Synovial sarcoma (SS) is a malignant mesenchymal neoplasm that commonly occurs in older children and young adults and various treatment, including surgery, chemotherapy and radiotherapy. BRCA2 is involved in DNA repair process and functions as a tumor suppressor gene, and aberrant BRCA2 promotes tumor growth. Currently, few studies have reported PSS with BRCA2 mutation and the therapy is still ambiguous.^12^, ^13^ Here, we first present a PSS patient who experienced disease relapse, BRCA2 mutation was identified by next‐generation sequencing (NGS) and had a robust response to pazopanib plus nivolumab with PFS of 14 months. This case suggests that BRCA2 mutation might be the molecular driver of a subset of PSS and a combination of immunotherapy and targeted therapy may be a potential option for these patients.

Pazopanib, a multitargeted TKI that predominantly inhibits vascular endothelial growth factor receptors (VEGFR) and platelet‐derived growth factor receptors (PDGFR), has been shown to increase immune cell infiltration of the tumor microenvironment by normalization of the tumor vasculature.^14^ Nivolumab, an inhibitor of programmed cell death receptor‐1 (PD‐1), has shown strong antitumor activity and improved survival outcome regardless of PD‐L1 expression level in non‐small cell lung cancer.^15^, ^16^, ^17^, ^18^ Some researchers believe nivolumab could alleviate inhibitory T cell signaling independently of tumoral PD‐L1 expression; for example, by blocking interactions with other PD‐1 ligands such as PD‐L2.^19^, ^20^, ^21^ The patient in our study was treated with pazopanib plus nivolumab with a partial response of up to 14 months, although with negative PD‐L1 expression. We speculate that pazopanib plus nivolumab caused a synergistic effect by increasing immune cell infiltration and facilitating immunotherapy response.

As this is only a single case report, more clinical studies are needed to explore the mechanism driving PSS and validate the efficacy of a combination of pazopanib and nivolumab in patients with PSS. The present study is also limited by the lack of an in vitro functional assay for BRCA2 and its sensitivity profile to BRCA inhibitors and various combination therapy based on immunotherapy.

In conclusion, we first present the good efficacy of pazopanib plus nivolumab in a PSS patient harboring a BRCA2 c.968dupT mutation. NGS is also supposed to be applied for subsequent treatment in patients with rapid disease progression.

## AUTHOR CONTRIBUTIONS

Xing Zhang collected the clinical data. Yongchang Zhang did the study design, data analysis, and data interpretation. Yongchang Zhang provided the pathology figure, wrote the first version of the article. Qinqin Xu contributed to the critical review and finalized the report. Written informed consent to publication was obtained.

## CONFLICT OF INTEREST STATEMENT

All authors declare no conflicts of interest.
